# On-target temporal characterization of optical pulses at relativistic intensity

**DOI:** 10.1038/s41377-019-0207-1

**Published:** 2019-10-23

**Authors:** Vyacheslav E. Leshchenko, Alexander Kessel, Olga Jahn, Mathias Krüger, Andreas Münzer, Sergei A. Trushin, Laszlo Veisz, Zsuzsanna Major, Stefan Karsch

**Affiliations:** 1 Max-Planck-Institut für Quantenoptik, 85748 Garching, Germany; 2 Department für Physik, Ludwig-Maximilians-Universität München, 85748 Garching, Germany; 3 Department of Physics, Umeå University, Umeå, SE-901 87 Sweden; 4 Present Address: Department of Physics, The Ohio State University, Columbus, OH 43210 USA; 5 GSI Helmholtzzentrum für Schwerionenforschung GmbH, Planckstraße 1, 64291 Darmstadt, Germany; 6 Helmholtz-Institut Jena, Fröbelstieg 3, 07743 Jena, Germany

**Keywords:** Nonlinear optics, High-harmonic generation, Ultrafast photonics, Laser-produced plasmas

## Abstract

High-field experiments are very sensitive to the exact value of the peak intensity of an optical pulse due to the nonlinearity of the underlying processes. Therefore, precise knowledge of the pulse intensity, which is mainly limited by the accuracy of the temporal characterization, is a key prerequisite for the correct interpretation of experimental data. While the detection of energy and spatial profile is well established, the unambiguous temporal characterization of intense optical pulses, another important parameter required for intensity evaluation, remains a challenge, especially at relativistic intensities and a few-cycle pulse duration. Here, we report on the progress in the temporal characterization of intense laser pulses and present the relativistic surface second harmonic generation dispersion scan (RSSHG-D-scan)—a new approach allowing direct on-target temporal characterization of high-energy, few-cycle optical pulses at relativistic intensity.

## Lasers: Lighting up high-intensity pulses

A new approach for characterizing intense optical pulses, by directly analyzing their interaction with a target, will help researchers to advance many strong-field applications. The development of applications such as laser-based charged-particle acceleration and bright coherent X-ray generation requires accurate knowledge of the characteristics of laser light at relativistic intensity. The approach is a refined form of ‘dispersion scanning’, which monitors the effect of an intense laser pulse as it interacts with material, thereby taking advantage of the processes that occur at relativistic intensities. The technique has been developed by Vyacheslav Leshchenko and colleagues at the Max-Planck Institute for Quantum Optics in Garching, Germany. It is especially effective at tackling the difficult challenge of characterizing how very short intense pulses vary with time.

## Introduction

Accurate knowledge of the on-target pulse intensity is essential for the correct interpretation of high-field experiments involving highly nonlinear processes. The on-target intensity can be estimated directly via different gas ionization processes, such as the intensity scaling of the above-threshold ionization^1,2^, the yield of single or multiple charged ions^3,4^, the photoelectron/photoion momentum distribution from ionization by circularly polarized laser fields^5^ and similar. However, these techniques are limited to intensities significantly below the relativistic limit ($1.37 \times 10^{18}\;{\mathrm{W}}/{\mathrm{cm}}^2$ at a central wavelength of 1 µm) and require a precise theory of the ionization process as well as knowledge of the temporal and spatial pulse shape to achieve reasonable precision of the averaging of the electron/ion impact over the intensity distribution^5^. Therefore, in high-intensity systems, the value of the on-target intensity is commonly estimated from the spatial and temporal power/fluence distributions, which are measured separately^6,7^. While there are reliable approaches for the detection of pulse energy and spatial distribution^8^, precise characterization of the on-target temporal structure remains a challenge, especially for intense, few-cycle pulses.

Achieving reliable detection of the spatial intensity distribution is simpler than achieving that of the temporal distribution, as it can be done directly with a CCD camera in combination with a microscope objective^8^, and the result is not affected by dispersive optical elements in the beam path (at least when they are of good enough quality and the beam is properly attenuated to prevent nonlinear distortions). One should note that a reliable measurement requires a high dynamic range (HDR) image to detect the energy contained in a possible background around the main peak. This image can be obtained with a combination of an HDR CCD camera and the additional extension of the dynamic range by the sequential measurement of unsaturated and saturated images with different attenuations.

In contrast, the temporal characterization of ultrashort optical pulses is always indirect and suffers from dispersive optics in the diagnostic beam path. It can be performed using different techniques involving reconstruction algorithms, such as FROG (frequency-resolved optical gating)^9^, SPIDER (spectral phase interferometry for direct electric-field reconstruction)^10^, a dispersion scan^11^, spatially resolved Fourier transform spectrometry^12^, and their modifications. The HDR temporal characterization is as important as the HDR focal beam profile diagnostics. Common and reliable methods of measuring the HDR temporal structure and determining the energy content in the main peak are third-order autocorrelation^13^, cross-correlation^14^ and self-referenced spectral interferometry, which allows single-shot implementation^15^. Note that the temporal contrast diagnostics and the pulse duration diagnostics are complementary. The former determines the energy content in the main pulse as well as in pre- and post-pulses and in the background but has poor temporal resolution (in the best cases, ~20–50 fs). Therefore, it cannot be used for the peak power evaluation of pulses with durations below ~100 fs. Thus, pulse duration diagnostics is required for the complete measurement of the temporal structure and corresponding peak power.

The problem of most pulse duration characterization approaches is that they cannot be applied at high intensities, which makes their results not always representative for the full-energy pulses. In addition to the difficulty of attenuation itself, the problem here lies in the dispersion, which is introduced in beamlines between the attenuation and diagnostics. Usually, it is necessary to split the beam somewhere before the target, strongly attenuate it, and guide it outside a vacuum chamber through a window, as most diagnostic devices are not vacuum compatible or require fine alignment, which makes their in-vacuum realization extremely complicated. All these procedures normally introduce some dispersion that cannot always be precisely traced back or post-compensated for accurate evaluation of the on-target pulse duration. For multi-cycle pulses (>10 fs pulse duration at ~1 µm central wavelength), generated, for example in Ti:Sa laser systems, this uncertainty is often tolerable. However, it is certainly not tolerable for few-cycle pulses generated in the emerging high-peak-power OPCPA (optical parametric chirped pulse amplification) systems^16–18^. Thus, many high-field experiments, especially with these systems, will profit from a direct on-target temporal characterization technique compatible with relativistic intensities ^19,20^.

A suitable approach can be a known temporal characterization technique involving second or higher-order harmonic generation, with the harmonic source being based on the relativistic surface high harmonic generation instead of a nonlinear crystal. Relativistic surface harmonics^21,22^ are compatible with very high pulse intensity and thus fulfill the main goal. There is, for example, a theoretical proposal of implementing an autocorrelator based on the detection of the third harmonic at relativistic intensities^20^. The temporal characterization approach to be used should ideally be reliable, robust and easy to implement but at the same time should be accurate and support ultrashort pulses. The dispersion scan (D-scan)^11^ method fulfills all these requirements and thus seems to be one of the best candidates. D-scan is based on the measurement of the dependence of the second harmonic spectrum on the varying dispersion in the fundamental beam path. Unlike autocorrelation approaches^19,20^, it requires no beam splitting and delaying of pulse replicas relative to each other and has been demonstrated to be suitable for the accurate characterization of few-cycle pulses^11,23^. Since our approach uses the D-scan scheme combined with relativistic surface second harmonic generation (SHG), we call it the relativistic surface second harmonic generation dispersion scan (RSSHG-D-scan).

**Fig. 1:**
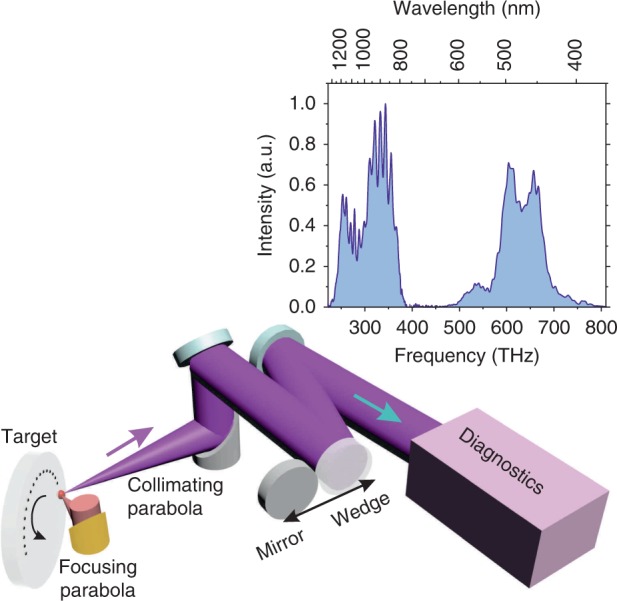
Scheme of the experimental setup. The p-polarized pulses are focused onto a BK7 target with a gold-coated ø1ʺ f/1.6 off-axis parabolic mirror. The reflected fundamental radiation and the generated harmonics are re-collimated with an aluminum-coated ø2ʺ f/1.3 off-axis parabolic mirror and guided with a set of mirrors and wedges outside the vacuum chamber for diagnostics. The inset shows a typical measured spectrum

This paper introduces the RSSHG-D-scan (Fig. 1). We demonstrate both experimental and PIC (particle in cell) simulation results, proving the feasibility of the technique, and discuss optimum experimental parameters and limitations of the method.

## Results

### SHG saturation intensity

**Fig. 2:**
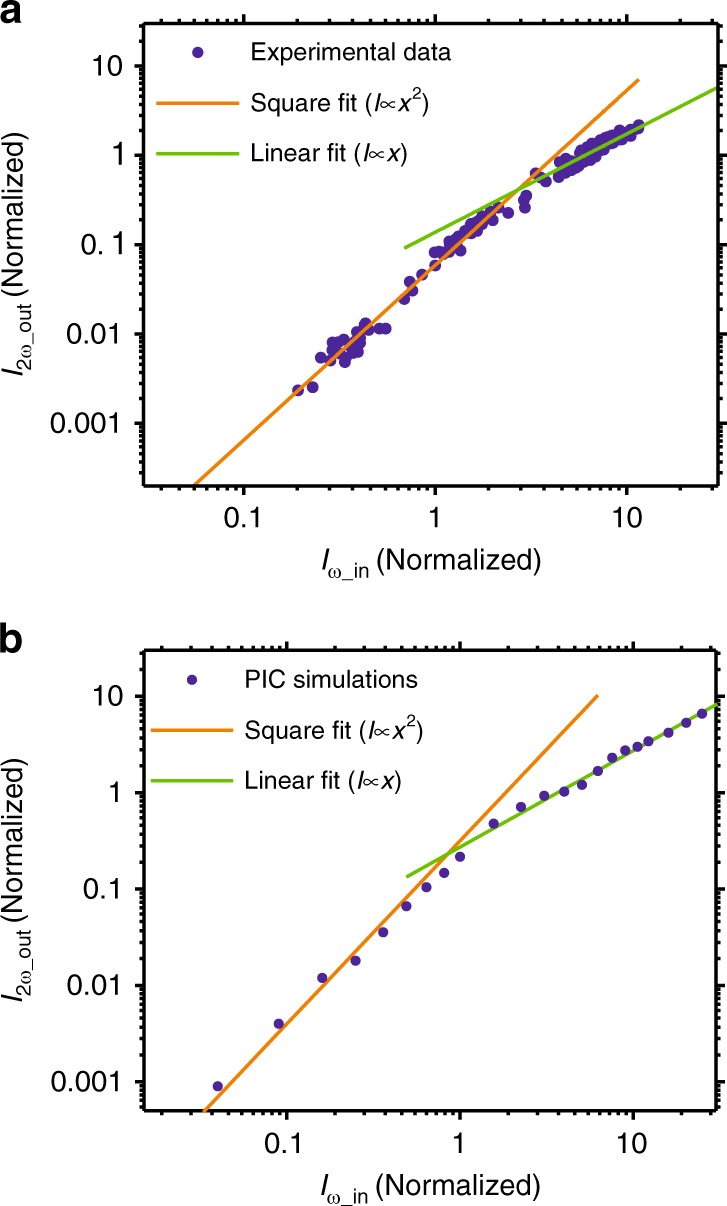
Nonlinearity of the RSSHG process. Experimental (**a**) and PIC simulation (**b**) results show very good agreement with each other. The simulation is performed for the plasma scale length of 0.2*λ*. The axes are normalized to $I_{\omega \_{\mathrm{in}}}(a_0 = 1)$; thus, the *x*-axis is $I_{\omega \_{\mathrm{in}}}/I_{\omega \_{\mathrm{in}}}(a_0 = 1)$, and the *y*-axis is $I_{2\omega \_{\mathrm{out}}}/I_{\omega \_{\mathrm{in}}}(a_0 = 1)$. The corresponding vector potential is $a_0 = \sqrt {I_{\omega \_{\mathrm{in}}}/I_{\omega \_{\mathrm{in}}}(a_0 = 1)}$, and $I_{\omega \_{\mathrm{in}}}(a_0 = 1)$ is the intensity corresponding to *a*_0_(*ω*) = 1. Both the experimental and simulation results demonstrate quadratic scaling (orange lines) for the normalized vector potential of the incoming field of $a_0 \,{\lesssim}\, 1$; however, for higher incoming intensities ($a_0 \,{\gtrsim}\, 2$), the SHG saturates, and the ratio becomes linear (green lines)

Saturation must be avoided in any measurement setup, including the RSSHG-D-scan. In a standard D-scan, SHG saturation needs to be avoided to ensure the quadratic scaling of the second harmonic signal with the intensity of the fundamental radiation, which allows for precise pulse reconstruction. This is normally automatically ensured by using a very thin nonlinear crystal, which is already required for broadband phase matching. In a RSSHG-D-scan, saturation of the second harmonic signal needs to be avoided as well. To determine the intensity range with quadratic SHG scaling, we measured the dependence of the generated second harmonic signal on the intensity of the incoming pulse (Fig. 2a). The experimental results demonstrate the quadratic scaling of the SHG efficiency for the normalized vector potential of an incoming field of $a_{0} \,{\lesssim}\, 1$ (see Methods for determination and more information); however, for higher intensities, the SHG saturates, and the ratio becomes linear. This experimental finding is in good agreement with the results of 1D PIC simulations^24^ (Fig. 2b), where both quadratic and linear scaling regions as well as the turning point between them are well reproduced.

A D-scan measurement at intensities in the saturation region results in a trace that cannot be accurately retrieved by a standard algorithm since it does not take saturation into account and thus fails. The simulated dependence of the reconstruction accuracy on the incoming pulse intensity and thus on the saturation level is presented in Table 1. The simulations were performed for *L*_p_ = 0.05*λ*. According to these data, increasing the incoming field strength over *a*_0_ = 1 causes an increase in the peak intensity reconstruction error. There are a few approaches to overcome this problem and to implement the suggested technique in an ultra-relativistic laser system. One option is a modification of the retrieval approach by taking SHG saturation into account, but this requires substantial theoretical support and will have problems with unambiguity of the retrieval result, especially at very high intensities (*a*_0_⨠1). Therefore, a better option is a well-controlled and characterized scaling down of the on-target intensity in the characterization setup. This can be realized, for example, by measuring the RSSHG-D-scan signal in the focus of another parabolic mirror with a longer focal length, or the incoming beam can be attenuated with a reflective wedge (or a set of wedges) to fulfill the condition of $a_0 \lesssim 1$. Unlike other contemporary temporal characterization approaches, the diagnostics can be implemented directly in a target chamber without an additional beamline, which would introduce dispersion.

**Table 1:** Simulated dependence of the pulse reconstruction accuracy on the peak intensity of the incoming pulse and thus on the SHG saturation level (Fig. 2)

*a*_0_	RMS phase reconstruction error (rad)	Peak intensity reconstruction error (%)
0.5	0.05	0.8
1	0.08	1.2
2	0.14	11
5	0.25	25
10	0.36	30

The analysis involves PIC simulations of high harmonic generation and the following D-scan reconstruction. The phase and intensity errors show the difference between the retrieved pulse parameters and the input of the PIC simulation. The root mean squared (RMS) phase error is calculated by weighting with the normalized spectral intensity (*I*_*ω*_) distribution: $\sqrt {\langle (I_\omega {\mathrm{\Delta }}\varphi - \langle I_\omega {\mathrm{\Delta }}\varphi \rangle )^2\rangle }$, where ${\mathrm{\Delta }}\varphi = \varphi _{{\mathrm{retrieved}}} - \varphi _{{\mathrm{input}}}$ and $\langle \rangle$ denotes the averaging. To simulate a realistic experimental scenario, the measured spectral intensity and phase, which are presented below in the Experimental Results section in Fig. 5c, are used as the input pulse parameters for the PIC simulations.

Another outcome of the presented results (Fig. 2) is the SHG efficiency optimum being at *a*_0_ ≈ 2–3. A further increase in the intensity will not improve the SHG efficiency, but it will result in energy transfer into higher harmonics, which saturate later^25^.

### Simulation results

**Fig. 3:**
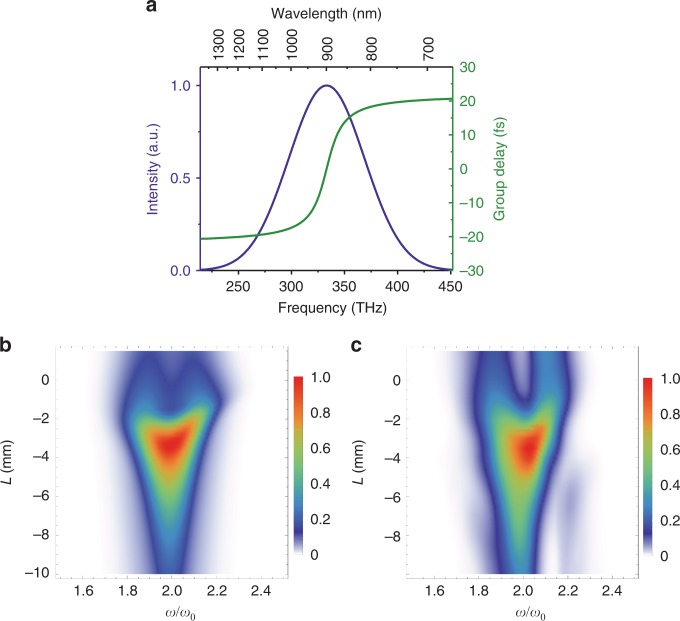
Theoretical proof of the feasibility of the RSSHG-D-scan by comparing it with the conventional SHG-D-scan method in simulation. **a** Spectral intensity and phase (in terms of group delay) used in the simulations. **b** Simulation of the SHG-D-scan (see main text for details). **c** PIC simulation of the RSSHG-D-scan (simulation parameters: *a*_0_ = 1 for the best compressed pulse, *L*_p_ = 0.05*λ*). *L* is the thickness of the fused silica, determining the dispersion introduced in the fundamental pulse

The theoretical feasibility of the RSSHG-D-scan was numerically investigated by comparing the RSSHG-D-scan with the standard SHG-D-scan using an input pulse with a nontrivial spectral phase (Fig. 3a). The standard SHG-D-scan was performed assuming perfect phase-matching conditions and using the following equation:

1 $$I_{2\omega }(\omega ,L) = \left| {{\int} {E_{2\omega }(t,L)e^{ - i\omega t}dt } } \right|^2$$

with

$$E_{2\omega }(t,L) = \left( {{\int} {E_\omega (\omega )e^{i\omega t + iLk(\omega )}d\omega } } \right)^2$$

where *E*_2*ω*_ and *I*_2*ω*_ are the field and intensity of the second harmonic signal, respectively, *E*_*ω*_ is the spectral field distribution of the incoming fundamental signal, *L* is the thickness of the material that is being scanned, and *k*(*ω*) = *n*(*ω*)*ω/c* is the wave vector, with *n*(*ω*) being the material dispersion. The RSSHG-D-scan was simulated with a 1D PIC code^24^. Both results, i.e., the second harmonic spectral intensity as a function of the inserted material length, are shown in Fig. 3b, c and demonstrate a very good agreement with the RMS difference of just *G* = 0.03 (see Methods section for definition), which represents a good level of consistency^26^. This consistency infers that all retrieval algorithms developed for the standard D-scan are directly applicable to the RSSHG-D-scan. Therefore, the RSSHG-D-scan method can directly profit from the advances in the retrieval approaches of the D-scan technique that have been developed over the last decade and are very well established.

### Experimental results

**Fig. 4:**
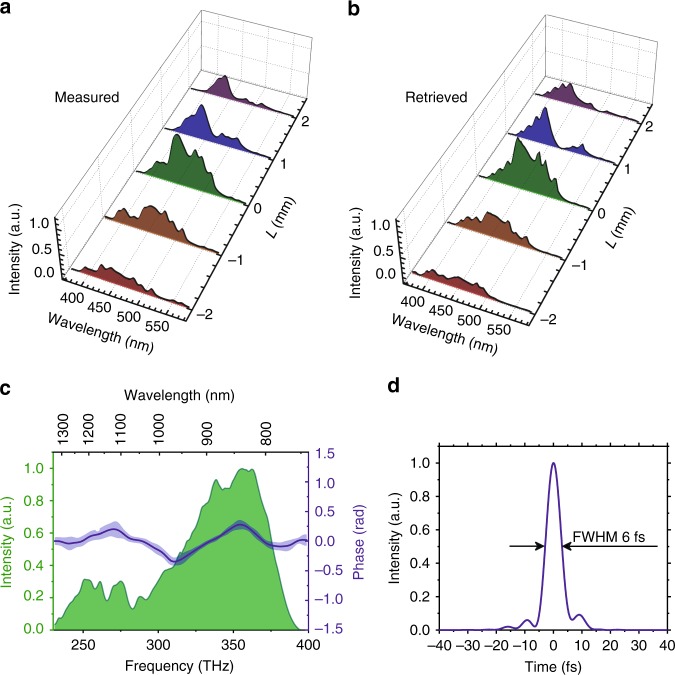
Experimental results of the SHG-D-scan using a 5-µm-thick BBO crystal. Measured (**a**) and retrieved (**b**) trace. The dispersion was changed by varying the thickness (*L*) of the fused silica substrates, which is measured relative to an arbitrarily defined 0 position, with smaller values corresponding to less material. **c** Retrieved spectral phase. The light-blue area around the main curve represents the uncertainty of the reconstructed spectral phase determined by the standard deviation of the retrieved function. **d** Reconstructed temporal pulse shape

**Fig. 5:**
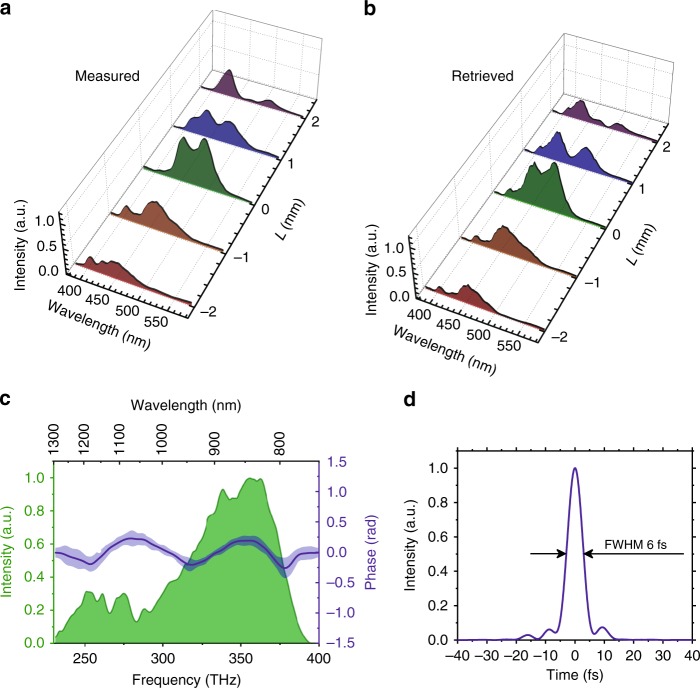
Experimental results of the RSSHG-D-scan. Measured (**a**) and retrieved (**b**) trace. The intensity of the best compressed pulse in the experiment corresponds to *a*_0_ ≈ 1. The dispersion was changed by varying the thickness (*L*) of the fused silica substrates, which is measured relative to an arbitrarily defined 0 position, with smaller values corresponding to less material. **c** Retrieved spectral phase. The light-blue area around the main curve represents the uncertainty of the reconstructed spectral phase determined by the standard deviation of the retrieved function. The shape of the phase resembles the optical parametric phase^16^; thus, it is caused by a slight mismatch between the final experimental parameters and the estimated parametric phase included in the design dispersion of the chirped mirrors. **d** Reconstructed temporal pulse shape. The retrieved full width at half maximum (FWHM) pulse duration is just 2% larger than the transform-limited one

The experimental test of the RSSHG-D-scan was performed using the setup schematically shown in Fig. 1 and described in more detail in the Methods section. To demonstrate the consistency of the RSSHG-D-scan with the standard SHG-D-scan, we characterized the same pulse using both techniques in parallel. As mentioned in the introduction, the measurement of the direct on-target pulse duration with a SHG-D-scan setup typically requires more effort. In particular, any dispersive medium needs to be avoided, which means that the setup has to be completely in vacuum and that no dispersive optical elements should be used. For this reason, only uncoated or metal-coated reflective optics were used in the implemented SHG-D-scan diagnostic setup. Second harmonic generation was performed in the thinnest commercially available BBO (β barium borate) crystal, with a 5 µm thickness, to ensure very good broadband phase matching. The RSSHG-D-scan, on the other hand, requires no special precautions, apart from the use of appropriate optics in the diagnostic beamline, which have no strong absorption in the spectral range of interest. The obtained experimental results are presented in Figs. 4 and 5. The relatively rough step size in the scan was mainly determined by technical issues caused by the necessity of re-optimization of the OPCPA stages for each material thickness (see Methods section for more details).

The retrieval approach based on the differential evolution algorithm^26^ was used. The RMS reconstruction error in both cases was 0.04. The uncertainty of the reconstructed spectral phase (Figs. 4c and 5c) determined by the standard deviation of the retrieved phase corresponds to the pulse duration uncertainty of 1%. The retrieved phases are in good agreement with each other as well as with the SHG-FROG results of our previous work on the laser system itself^16^. Note that this latter result has an opposite sign due to the sign ambiguity of the SHG-FROG method. The shape of the residual spectral phase resembles the optical parametric phase^16^ and is most likely caused by the matching of the dispersion of the chirped mirror compressor to the entire system, which is quite well optimized, but it is not perfect.

Although, as shown above, it is possible (in principle) to perform a reliable measurement with the SHG-D-scan, it has many drawbacks compared with the RSSHG-D-scan. First, the RSSHG-D-scan is a more direct technique, ensuring that the pulse duration is measured directly on-target. Second, the efficiency of the second harmonic generation in a thin crystal is small, causing detection problems, especially in the presence of a parasitic SHG background. For example, nonlinear crystals used in OPA systems quite often support some collinear second harmonic generation that contains not more than a fraction of a percent of the total energy of the amplified pulse and thus causes no problems for experiments, but this parasitic SHG signal from a few mm-thick OPA crystals can be higher than the SHG-D-scan signal from the few µm-thick diagnostic crystal needed for good phase matching. As will be discussed in more detail below, RSSHG shows a very high conversion efficiency, which makes it very tolerant to any parasitic background. Note that the problem of the parasitic SHG background can be avoided in a standard D-scan using the cross-polarized wave XPW-D-scan^27^, but this approach suffers from other problems, such as dispersion of the polarizers and polarization purity of the incoming beam. Third, a potential error in the estimation of the introduced dispersion will result in a pronounced increase in the reconstruction error, which will indicate that something is wrong. In contrast, in any out-of-vacuum diagnostic approach, an incorrect estimate of the beamline dispersion directly affects the measured pulse duration without any indication of the inconsistency. In addition, in our setup, the RSSHG-D-scan was simpler to design, build and align than the classical SHG-D-scan setup.

### RSSHG conversion efficiency

RSSHG can support high conversion efficiency for ultrashort high-energy pulses^28^, as one can directly see from the typical detected spectrum presented in Fig. 1. At the optimum pre-plasma scale length, the integrated detected energy in the reflected beam in the second harmonic range was of the same order as in the fundamental range. In particular, the measured energy was 5 mJ for both the fundamental and second harmonic beams after taking into account the beamline transmission. An FEL700 filter was used to measure the fundamental energy, and a BG40 filter, to measure the second harmonic. The incoming pulse energy was 22 mJ, which is equivalent to *a*_0_ ≈ 3. Thus, the measured energies correspond to 23% of the second harmonic conversion efficiency. The rest of the incoming energy is partially converted into higher harmonics that are outside the detection range of the used spectrometers and might be partially absorbed or transmitted by the target.

The experimentally demonstrated high SHG conversion efficiency is quite attractive for many applications requiring broadband high-energy second or even higher-order harmonic radiation^29–33^, especially in combination with their good phase-locking properties^34,35^. For example, RSSHG can be used for the generation of very short optical wave forms by synthesizing the fundamental radiation with the generated second harmonic and potentially higher harmonics in a way similar to the generation of light transients from a supercontinuum after hollow-core fibers ^30^.

**Fig. 6:**
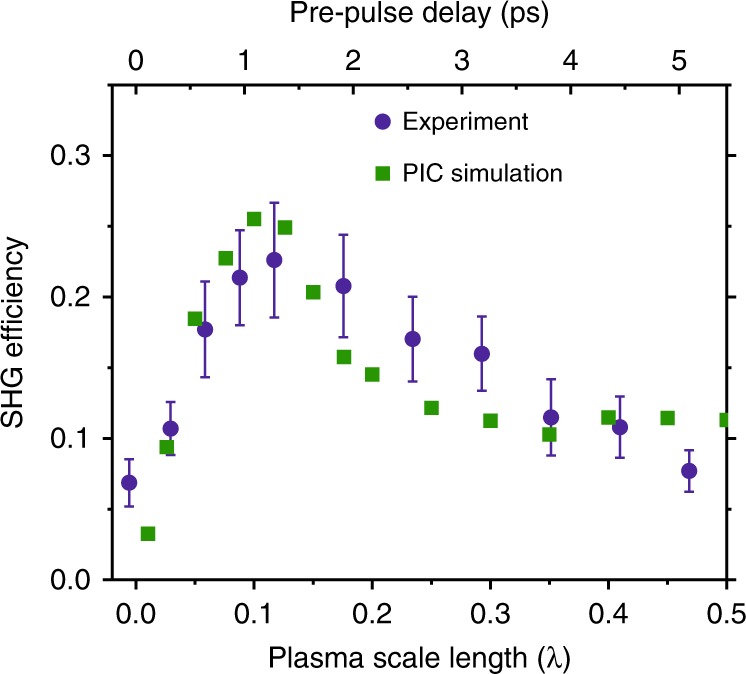
Measured (blue round dots) and simulated (green squares) dependence of the SHG efficiency on the pre-pulse delay and corresponding plasma scale length. SHG efficiency is determined as $U_{2\omega \_{\mathrm{out}}}/U_{\omega \_{\mathrm{in}}}$, with $U_{\omega \_{\mathrm{in}}}$ being the incoming fundamental energy and $U_{2\omega \_{\mathrm{out}}}$ being the reflected energy in the SHG spectral region. Error bars originate from the standard deviation of multiple measurements. The results demonstrate the excellent agreement between the experiment and simulation and have a clear optimum at the plasma scale length of *L*_p_ ≈ 0.1*λ*

The SHG conversion efficiency depends on the pre-plasma scale length, as was demonstrated for higher harmonics in other experiments on relativistic surface high harmonic generation^36–40^, and the 23% efficiency presented above was achieved only under optimum conditions. The performed pre-pulse scan (Fig. 6) shows a clear optimum around the plasma scale length of $L_{\mathrm{p}} \approx 0.1\lambda - 0.15\lambda$, which agrees well with the predictions of the PIC simulations (Fig. 6), having a maximum at $L_{\mathrm{p}} \approx 0.1\lambda$. Therefore, the experiments, aiming at efficient frequency doubling, should be performed at a plasma scale length of ~0.1*λ*.

## Discussion

We have presented RSSHG-D-scan—a technique for the direct on-target temporal characterization of high-energy, few-cycle optical pulses at relativistic intensity. The feasibility of the approach for the temporal characterization of few-cycle pulses was proved both experimentally and in PIC simulations. The direct on-target temporal characterization of 6 fs pulses with few-TW peak power was experimentally demonstrated. RSSHG-D-scan will be especially suitable for pulse characterization in emerging high-energy, few-cycle OPCPA-based systems and can play an important role in the optimization of pulse compression in applications such as high-order harmonic generation and laser-based particle acceleration.

In addition, RSSHG can be used for efficient second harmonic generation. A conversion efficiency of 23% was experimentally demonstrated, which is in good agreement with the results of PIC simulations. This high SHG conversion efficiency offers possibilities for many applications, including the synthesis of single-cycle optical pulses at an energy level that has not been accessible thus far.

## Materials and methods

### Experimental setup

The scheme of the experimental setup is shown in Fig. 1. The relativistic surface harmonics are driven by the pulses generated in the OPCPA system, providing a 6–7 fs pulse duration at a 900 nm central wavelength with up to 25 mJ of energy on a target and a contrast of better than 10^11^ at a rise time of only 1 ps^16^. The typical (RMS) pulse energy instability was 3%. As demonstrated in previous works^36–40^, the performance of relativistic surface harmonics is very sensitive to the pre-plasma gradient, which needs to be optimized. To do so, a pre-pulse with an intensity of ~10^15^ W/cm^2^ and an adjustable delay (*τ*) is introduced before the main pulse^36^ to pre-ionize the target and initiate the plasma expansion with a speed of *C*_s_ ≈ 80 nm/ps^41^, leading to an approximately exponential^42^ plasma density gradient at the solid–vacuum interface ($N_{\mathrm{e}} = N_0e^{ - x/L_{\mathrm{p}}}$) with a scale length of *L*_p_ = *C*_s_ × *τ*. The radiation reflected from the target (including generated harmonics and the rest of the fundamental harmonic) is re-collimated with an f/1.3 90^o^ off-axis Al-coated parabolic mirror and guided out of the vacuum chamber, where the spectrum is recorded with a set of two spectrometers (NIRQuest-512 and HR2000 + from Ocean Optics) covering the entire fundamental and second harmonic spectral range.

All parts of the experimental setup, starting from the compressor of the pump pulses and the OPCPA stages, were in vacuum to prevent the degradation of the beam quality due to nonlinear distortions. The material thickness during the dispersion scan was changed before the seed pulse was coupled into the vacuum setup and therefore before the amplification chain. This resulted in a change in the delay between pump and seed pulses in the OPCPA stages, which needed to be re-optimized for each material thickness to ensure identical parameters of the amplified pulses. This was the main factor limiting the number of steps in the dispersion scan during one experimental campaign. Note that the above-mentioned problem is specific to our current beamline design and not a general one. Therefore, it can be solved in our setup after the next upgrade, and it can be avoided in other systems. In particular, wedges for the variation of the material thickness can be installed after the OPCPA (but preferably before the compressor).

During the dispersion scan, each spectrum was measured by averaging over 10 laser shots to diminish the influence of the pulse energy instability.

### Effective vector potential evaluation

The laser parameters listed above correspond to a peak intensity of $I = 4 \times 10^{19}\;{\mathrm{W}}/{\mathrm{cm}}^2$ resulting in the maximum normalized vector potential of $a_0\,=\,4.8$, where $a_0 = eE_0/m_{\mathrm{e}}\omega c = \sqrt {I\lambda ^2/(1.37 \times 10^{18})}$, $\lambda$ is the central wavelength in µm, $I$ is the intensity in W/cm^2^, *e* and $m_{\mathrm{e}}$ are the electron charge and mass, respectively, and *E*_0_ and *ω* are the laser field amplitude and angular frequency, respectively. However, to compare the experimental results with the 1D PIC simulations, an effective normalized value, in our case, averaged over the full width at half maximum diameter of the focal intensity distribution, should be used. The maximum effective vector potential in the performed experiments was thus $\approx 4.8/\sqrt 2 = 3.4$, the second power of which limits, for example, the maximum value of $I_{\omega \_{\mathrm{in}}}$ in Fig. 2a.

### PIC simulations

1D PIC simulations were performed with the LPIC + +^24^ code. In the simulations, the plasma density profile has an exponential layer in front of a constant slab density layer. The density of the interface layer rises from 0.1*n*_c_ up to a maximum of 400*n*_c_, corresponding to the density of a glass target when fully ionized, where *n*_c_ is the critical electron density. The p-polarized laser pulse is incident onto the plasma at an angle of 45°. This oblique incidence geometry is transformed into a 1D case using the Bourdier technique^43^.

### D-scan retrieval

The retrieval of the D-scan results presented in this paper was carried out with a home-written program based on the differential evolution algorithm and a reconstruction procedure described in detail elsewhere^26^.

The error of the D-scan retrieval was determined using the standard equation^26^:

$$G = \sqrt {\frac{1}{{NM}}\mathop {\sum }\limits_{i = 1}^N \;\mathop {\sum }\limits_{j = 1}^M \;I_{2\omega }^{({\mathrm{meas}})}\;\left( {\omega _i,L_j} \right) - R_iI_{2\omega }^{({\mathrm{retr}})}(\omega _i,L_j)}$$

where $I_{2\omega }^{({\mathrm{meas}})}$ and $I_{2\omega }^{({\mathrm{retr}})}$ are the measured/simulated and retrieved SHG signals, respectively, which are normalized to the unity maximum value, *N* and *M* are the resolution (length of the data list) in the spectral and dispersion domains, respectively, and R accounts for (in the case of measured data) the spectrometer response and SHG conversion efficiency:

$$R_i = \frac{{\mathop {\sum }\nolimits_{j = 1}^M \;I_{2\omega }^{({\mathrm{meas}})}(\omega _i,L_j)I_{2\omega }^{({\mathrm{retr}})}(\omega _i,L_j)}}{{\mathop {\sum }\nolimits_{j = 1}^M \;I_{2\omega }^{({\mathrm{meas}})}\;\left( {\omega _i,L_j} \right)^2}}$$

## Supplementary information

Supplementary information

## Data Availability

The data that support the results presented in the paper are available from the corresponding authors upon reasonable request.

## References

[1] Pullen MG (2013). Measurement of laser intensities approaching 10^15^ W/cm^2^ with an accuracy of 1%. Phys. Rev. A.

[2] Wang Y (2009). Direct theoretical method for the determination of peak laser intensities from Freeman resonances in above-threshold ionization. Phys. Rev. A.

[3] Bhardwaj VR (2001). Few cycle dynamics of multiphoton double ionization. Phys. Rev. Lett..

[4] Peterson ER, Bucksbaum PH (2001). Above-threshold double-ionization spectroscopy of argon. Phys. Rev. A.

[5] Quan W (2016). Laser intensity determination using nonadiabatic tunneling ionization of atoms in close-to-circularly polarized laser fields. Opt. Express.

[6] Yanovsky V (2008). Ultra-high intensity-300-TW laser at 0.1 Hz repetition rate. Opt. Express.

[7] Kiriyama H (2018). High-contrast high-intensity repetitive petawatt laser. Opt. Lett..

[8] Bahk SW (2004). Generation and characterization of the highest laser intensities (10^22^ W/cm^2^). Opt. Lett..

[9] Trebino R (1997). Measuring ultrashort laser pulses in the time-frequency domain using frequency-resolved optical gating. Rev. Sci. Instrum..

[10] Iaconis C, Walmsley IA (1998). Spectral phase interferometry for direct electric-field reconstruction of ultrashort optical pulses. Opt. Lett..

[11] Miranda M (2012). Characterization of broadband few-cycle laser pulses with the d-scan technique. Opt. Express.

[12] Pariente G (2016). Space-time characterization of ultra-intense femtosecond laser beams. Nat. Photonics.

[13] Langlois P, Ippen EP (1999). Measurement of pulse asymmetry by three-photon-absorption autocorrelation in a GaAsP photodiode. Opt. Lett..

[14] Wang YZ (2014). Single-shot measurement of >1010 pulse contrast for ultra-high peak-power lasers. Sci. Rep..

[15] Oksenhendler T (2017). High dynamic, high resolution and wide range single shot temporal pulse contrast measurement. Opt. Express.

[16] Kessel A (2018). Relativistic few-cycle pulses with high contrast from picosecond-pumped OPCPA. Optica.

[17] Rivas DE (2017). Next generation driver for attosecond and laser-plasma physics. Sci. Rep..

[18] Budriunas R (2017). 53 W average power CEP-stabilized OPCPA system delivering 5.5 TW few cycle pulses at 1 kHz repetition rate. Opt. Express.

[19] Liesfeld B (2005). Single-shot autocorrelation at relativistic intensity. Appl. Phys. Lett..

[20] Guo Z (2018). Autocorrelation pulse-duration measurement of relativistic femtosecond laser. Phys. Plasmas.

[21] Quéré F (2006). Coherent wake emission of high-order harmonics from overdense plasmas. Phys. Rev. Lett..

[22] Baeva T, Gordienko S, Pukhov A (2006). Theory of high-order harmonic generation in relativistic laser interaction with overdense plasma. Phys. Rev. E.

[23] Fabris D (2015). Single-shot implementation of dispersion-scan for the characterization of ultrashort laser pulses. Opt. Express.

[24] Lichters R, Meyer-ter-Vehn J, Pukhov A (1996). Short-pulse laser harmonics from oscillating plasma surfaces driven at relativistic intensity. Phys. Plasmas.

[25] Tsakiris GD (2006). Route to intense single attosecond pulses. New J. Phys..

[26] Escoto E (2018). Advanced phase retrieval for dispersion scan: a comparative study. J. Optical Soc. Am. B.

[27] Tajalli A (2016). Few-cycle optical pulse characterization via cross-polarized wave generation dispersion scan technique. Opt. Lett..

[28] Hörlein R (2008). High contrast plasma mirror: spatial filtering and second harmonic generation at 10^19^ W cm^−2^. New J. Phys..

[29] Hänsch T (1990). A proposed sub-femtosecond pulse synthesizer using separate phase-locked laser oscillators. Opt. Commun..

[30] Wirth A (2011). Synthesized light transients. Science.

[31] Chen BQ (2015). High-efficiency broadband high-harmonic generation from a single quasi-phase-matching nonlinear crystal. Phys. Rev. Lett..

[32] Wang Y (2017). 0.85 PW laser operation at 3.3 Hz and high-contrast ultrahigh-intensity λ = 400 nm second-harmonic beamline. Opt. Lett..

[33] Edwards MR, Platonenko VT, Mikhailova JM (2014). Enhanced attosecond bursts of relativistic high-order harmonics driven by two-color fields. Opt. Lett..

[34] Nomura Y (2009). Attosecond phase locking of harmonics emitted from laser-produced plasmas. Nat. Phys..

[35] Quéré F (2008). Phase properties of laser high-order harmonics generated on plasma mirrors. Phys. Rev. Lett..

[36] Kahaly S (2013). Direct observation of density-gradient effects in harmonic generation from plasma mirrors. Phys. Rev. Lett..

[37] Rödel C (2012). Harmonic generation from relativistic plasma surfaces in ultrasteep plasma density gradients. Phys. Rev. Lett..

[38] Dollar F (2013). Scaling high-order harmonic generation from laser-solid interactions to ultrahigh intensity. Phys. Rev. Lett..

[39] Kormin D (2018). Spectral interferometry with waveform-dependent relativistic high-order harmonics from plasma surfaces. Nat. Commun..

[40] Jahn O (2019). Towards intense isolated attosecond pulses from relativistic surface high harmonics. Optica.

[41] Adumi K (2004). Characterization of preplasma produced by an ultrahigh intensity laser system. Phys. Plasmas.

[42] Crow JE, Auer PL, Allen JE (1975). The expansion of a plasma into a vacuum. J. Plasma Phys..

[43] Bourdier A (1983). Oblique incidence of a strong electromagnetic wave on a cold inhomogeneous electron plasma. Relativistic Eff. Phys. Fluids.

