# Supplementary material for: On-target temporal characterization of optical pulses at relativistic intensity
Source: Light Sci Appl. 2019 Oct 23;8:96. doi: 10.1038/s41377-019-0207-1 (PMC6813334; doi:10.1038/s41377-019-0207-1)
Supplement: Supplementary file 1 — Supplementary information [file 41377_2019_207_MOESM1_ESM.pdf]

Supplementary information

On-target temporal characterization of optical pulses at relativistic intensity

Vyacheslav E. Leshchenko[1,2,3,*], Alexander Kessel[1,2], Olga Jahn[1,2], Mathias Krüger[1,2], Andreas Münzer[1,2], Sergei A. Trushin[1,2], Laszlo Veisz[1,4], Zsuzsanna Major[1,2,5], and Stefan Karsch[1,2,*]

[1]Max-Planck-Institut für Quantenoptik, 85748 Garching, Germany
[2]Department für Physik, Ludwig-Maximilians-Universität München, 85748 Garching, Germany
[3]Present address: Department of Physics, The Ohio State University, Columbus, Ohio 43210, USA
[4]Department of Physics, Umeå University, Umeå, Sweden
[5]Present address: GSI Helmholtzzentrum für Schwerionenforschung GmbH, Planckstraße 1, 64291 Darmstadt, Germany & Helmholtz-Institut Jena, Fröbelstieg 3, 07743 Jena, Germany
[*]Correspondence and requests for materials should be addressed to V.E.L. (email: leschenkoslava@gmail.com) or S.K. (email: stefan.karsch@mpq.mpg.de).

ABSTRACT

This document provides supplementary information to "On-target temporal characterization of optical pulses at relativistic intensity". In this supplement, we provide additional technical details and simulation results supporting the paper which can also be useful for a successful implementation of the Relativistic Surface Second Harmonic Generation Dispersion scan (RSSHG-D-scan).

1 Averaging over the focal intensity distribution.

Since a typical far-field profile of a laser beam has a Gaussian like main peak (even for a flat-top near-field shape), a question of the averaging of the second harmonic (SHG) signal over a focal intensity distribution might arise. Results presented in Fig. S1 demonstrate that it is not an issue for the RSSHG-D-scan approach because spectral shape of the second harmonic signal has no significant dependence on the intensity of the fundamental field. There are no significant modifications of the spectral shape of the SHG signal even in saturation (for $a_0 > 1$) and the main reason for the degradation of the reconstruction accuracy (presented in Table 1 in the paper) is not a degradation of the SHG spectral shape but mainly the change of the SHG efficiency scaling (presented in Fig. 2 in the paper) from quadratic to linear. Therefore, the main requirement for a precise reconstruction is keeping the normalized vector potential corresponding to the pulse peak intensity below unity, the estimation of which requires an approximate knowledge of the focal spot. In summary, the averaging over the focal intensity distribution does not change the results presented in the paper.

2 Dependence of the RSSHG efficiency saturation on the plasma scale length.

Results on the dependence of the RSSHG saturation for the plasma scale length of $L_p = 0.2\lambda$ are shown in Fig. 2 in the main paper. Here, in Fig. S2, we provide additional simulation results for different plasma scale lengths. These results demonstrate the tendency of a slight increase of the saturation point from $a_0 \approx 1$ (or equivalently $I_{\omega\_in} \approx 1$) to about $a_0 \approx 1.5 - 2$ (or equivalently $I_{\omega\_in} \approx 3 - 4$) with increasing the plasma scale length. However, the shift of the saturation point is minor. Therefore, it doesn't influence the conclusion made in the paper that a precise reconstruction requires $a_0 \lesssim 1$.

The presented shift of the saturation point to higher intensities for larger pre-plasma scale lengths might lead to an idea that it can be used to increase the maximum applicable intensity of the presented RSSHG-D-scan approach. Unfortunately, it will not work due to degradation of the reconstruction accuracy with increasing the plasma scale length especially at high intensities as one can see from Fig. S3. This degradation of the reconstruction accuracy is caused by spectral distortions introduced by a too "soft" plasma with a large pre-plasma scale length similar to the distortions detected for higher harmonics[1].

Therefore the pre-plasma scale length should be not too large ($\lesssim 0.5\lambda$) ideally around $L_p \sim 0.05\lambda - 0.2\lambda$ for the succesfull implementation of the RSSHG-D-scan approach. Although it is basically sufficient to know that $L_p < 0.5\lambda$ for the application of the RSSHG-D-scan, a precise estimation of the reconstruction uncertainty might require a direct measurement of the plasma profile with one of the well-established techniques[2,3]. In summary, the additional results presented in this section do not change any conclusion made in the paper.

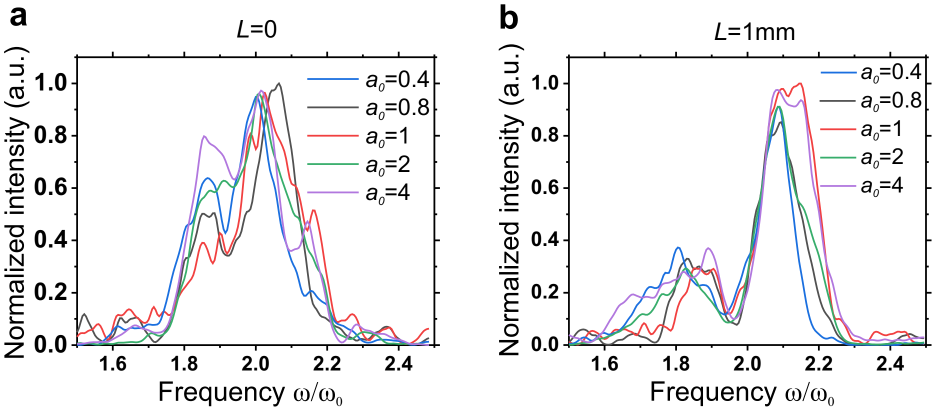

Figure S1. Simulated second harmonic spectra for different peak intensities of the characterized pulse for the dispersion corresponding to fused silica with zero (a) and 1 mm (b) thickness (L). Spectral intensity and phase presented in Fig 5c in the main paper were used as input fundamental field for the particle in cell (PIC) simulations.

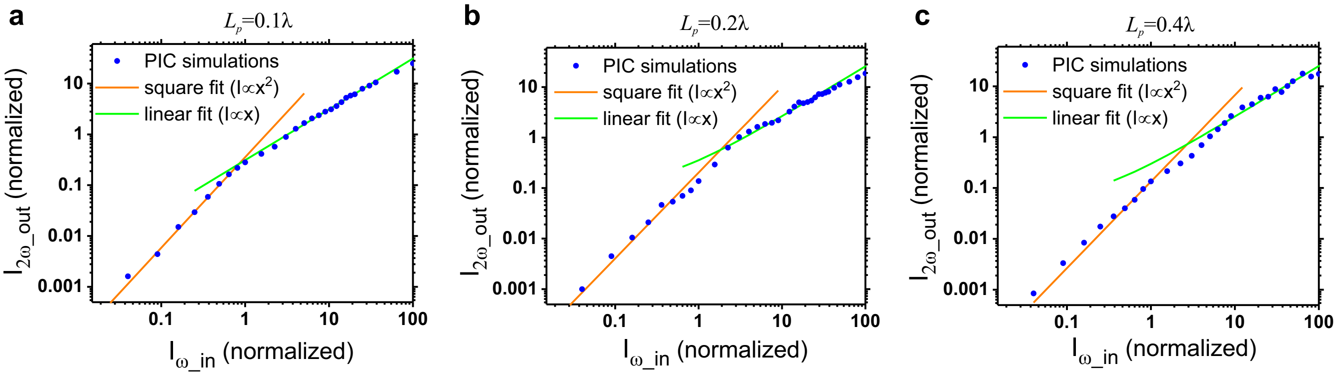

Figure S2. Simulated dependence of the nonlinearity of the RSSHG process for different pre-plasma scale lengths with otherwise identical simulation parameters to the results presented in Fig. 2b in the main text.

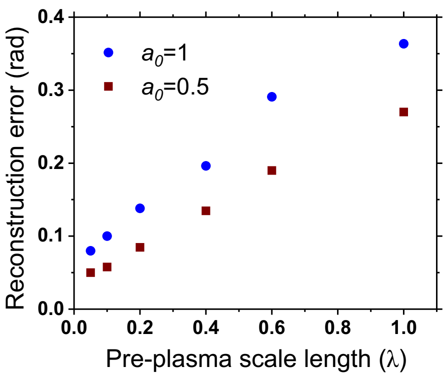

Figure S3. Simulation results on the dependence of the RMS spectral phase reconstruction error on the pre-plasma scale length. PIC simulation were done for the same pulse input parameters as in Table 1 in the main text.

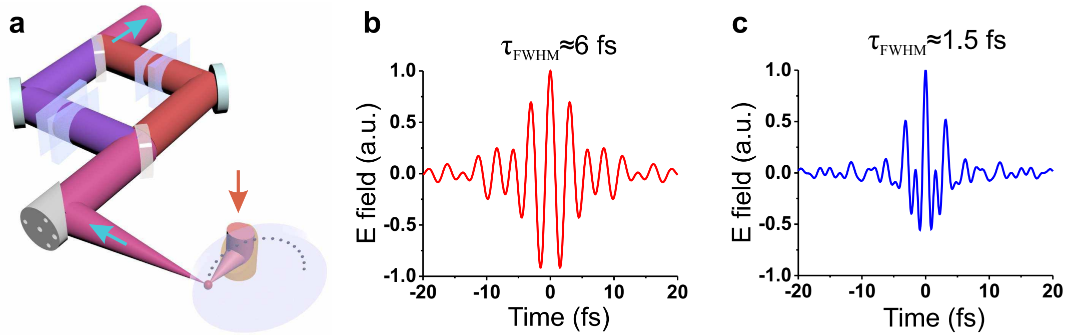

Figure S4. (**a**) Sketch of a waveform synthesizer based on the RSSHG approach. The reflected fundamental radiation and generated second harmonic signal are separated and, after the adjustment of the dispersion in each arm, recombined in a controlled way to synthesize an electric field showing a very short temporal structure. (**b**) The field of the incoming few-cycle pulse corresponding to the spectrum shown in Fig. 5c in the main paper. (**c**) The simulation of a possible synthesized single-cycle pulse corresponding to the transform-limited compression of the measured spectrum shown in Fig. 1 in the main paper.

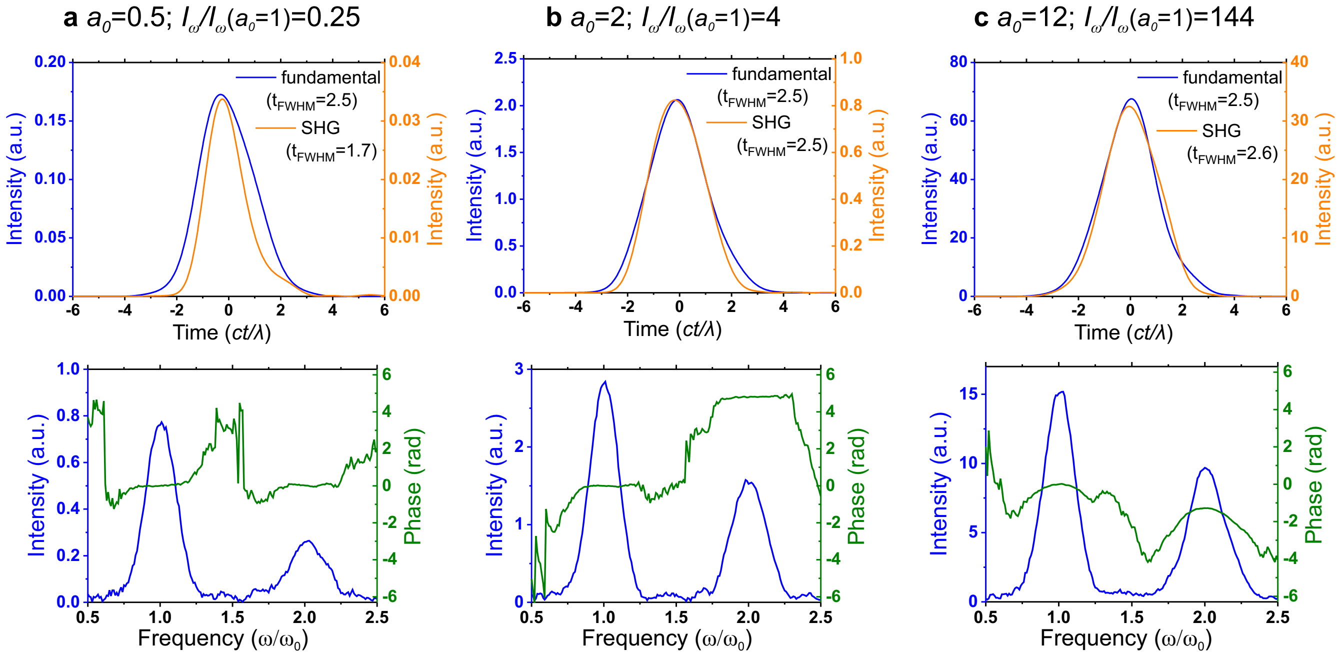

Figure S5. Simulated temporal structure (upper raw) and spectral intensity and phase (lower raw) of the reflected from the plasma mirror radiation for normalized peak intensity of 0.25, 4 and 144 (which corresponds to the normalized vector potential a_0 of 0.5, 2 and 12 accordingly). A transform limited Gaussian pulse with full width at half maximum (FWHM) duration of 2.5 optical cycles were used as an input for the simulations. A plasma scale length of 0.1 λ were used in simulations which corresponds to the optimum conversion efficiency, according to the results in the main text. The time units are optical cycles; the frequency axis is normalized to the central frequency of the fundamental pulse (ω_0).

3 Temporal structure of the reflected pulse.

In the main paper, we propose the application of the RSSHG for the generation of very short optical pulses at very high peak power by synthesizing the fundamental radiation with the generated second harmonic. Here, we provide additional simulation results supporting this idea. A basic scheme of such a synthesizer is shown in Fig. S4. The fundamental and second harmonic spectral parts of the reflected radiation are split with a dichroic beam splitter; the spectral phases of these pulses and the delay between them are optimized to provide the shortest recombined pulse, which for our experimentally registered spectra would support sub-cycle pulse duration (Fig. S4c). Since the pulse synthesis approach relies on the RSSHG in the saturation regime (due to the highest SHG efficiency), there is a question of the amount of temporal and spectral distortions introduced in

saturation. The simulations (Fig. S5) show that the situation is actually very similar to what one would expect from SHG in a nonlinear crystal with perfect phase matching. As presented in Fig. S5, before saturation (for $a_0 < 1$), the second harmonic pulse is approximately by the factor of $\sqrt{2}$ shorter than the fundamental one and the spectral phases are nearly flat; after approaching saturation ($a_0 \gtrsim 1$) the SHG pulse has nearly same shape and duration as the fundamental one and the spectral phases are still nearly flat; in strong saturation ($a_0 \gg 1$) durations are still nearly equal although slight mostly second order chirp appears. Since the RSSHG efficiency doesn't increase after approaching saturation, it is better to implement the pulse synthesis at $a_0 \sim 2-3$ where spectral phases are still flat, as it is suggested in the paper. Therefore, according to the performed simulations, RSSHG in saturation has no problems with degradation of the temporal shape or spectral phase of the reflected pulses, and it seems to be a promising basis for future high-power pulse synthesizers.

References

1. Behmke, M. *et al.* Controlling the spacing of attosecond pulse trains from relativistic surface plasmas. *Phys. Rev. Lett.* **106**, 185002 (2011).

2. Deneuville, F. *et al.* Sub-picosecond and nanometer scale dynamics of aluminum target surface heated by ultrashort laser pulse. *Applied Physics Letters* **102**, 194104 (2013).

3. Bocoum, M. *et al.* Spatial-domain interferometer for measuring plasma mirror expansion. *Opt. Lett.* **40**, 3009–3012 (2015).
